# Automatic differentiation of thyroid scintigram by deep convolutional neural network: a dual center study

**DOI:** 10.1186/s12880-021-00710-4

**Published:** 2021-11-25

**Authors:** Pei Yang, Yong Pi, Tao He, Jiangming Sun, Jianan Wei, Yongzhao Xiang, Lisha Jiang, Lin Li, Zhang Yi, Zhen Zhao, Huawei Cai

**Affiliations:** 1 Laboratory of Clinical Nuclear Medicine, Department of Nuclear Medicine, West China Hospital, Sichuan University, No.37 Guo Xue Alley, Chengdu, 610041 People’s Republic of China; 2 Machine Intelligence Laboratory, College of Computer Science, Sichuan University, Chengdu, 610065 People’s Republic of China; 3 Department of Nuclear Medicine, Panzhihua Central Hospital, Panzhihua, 617067 People’s Republic of China

**Keywords:** Artificial intelligence, Deep convolutional neural network, Thyroid scintigraphy

## Abstract

**Background:**

^99m^Tc-pertechnetate thyroid scintigraphy is a valid complementary avenue for evaluating thyroid disease in the clinic, the image feature of thyroid scintigram is relatively simple but the interpretation still has a moderate consistency among physicians. Thus, we aimed to develop an artificial intelligence (AI) system to automatically classify the four patterns of thyroid scintigram.

**Methods:**

We collected 3087 thyroid scintigrams from center 1 to construct the training dataset (n = 2468) and internal validating dataset (n = 619), and another 302 cases from center 2 as external validating datasets. Four pre-trained neural networks that included ResNet50, DenseNet169, InceptionV3, and InceptionResNetV2 were implemented to construct AI models. The models were trained separately with transfer learning. We evaluated each model’s performance with metrics as following: accuracy, sensitivity, specificity, positive predictive value (PPV), negative predictive value (NPV), recall, precision, and F1-score.

**Results:**

The overall accuracy of four pre-trained neural networks in classifying four common uptake patterns of thyroid scintigrams all exceeded 90%, and the InceptionV3 stands out from others. It reached the highest performance with an overall accuracy of 92.73% for internal validation and 87.75% for external validation, respectively. As for each category of thyroid scintigrams, the area under the receiver operator characteristic curve (AUC) was 0.986 for ‘diffusely increased,’ 0.997 for ‘diffusely decreased,’ 0.998 for ‘focal increased,’ and 0.945 for ‘heterogeneous uptake’ in internal validation, respectively. Accordingly, the corresponding performances also obtained an ideal result of 0.939, 1.000, 0.974, and 0.915 in external validation, respectively.

**Conclusions:**

Deep convolutional neural network-based AI model represented considerable performance in the classification of thyroid scintigrams, which may help physicians improve the interpretation of thyroid scintigrams more consistently and efficiently.

## Background

Thyroid scintigraphy with ^99m^Tc-pertechnetate is an essential complementary exanimation for the evaluation of thyroid function as a follow-up to blood biochemical tests and thyroid ultrasonography. It is a valid and convenient avenue to identify the causes of thyrotoxicosis, especially for distinguishing Graves’ disease (GD) and toxic multinodular goiter (TMG) when both thyrotropin receptor antibody was negative or differentiating GD from thyroiditis [1]. Accurate interpretation of thyroid scintigrams influences treatment decisions. If clinicians interpret the same scintigram differently, they will likely recommend different treatments. The interpretation of thyroid scintigram is always focused on the degree of radionuclide uptake, which was mostly described as diffuse or focal, homogeneous or heterogeneous, and increased or decreased [2]. Albeit, the interpretation of thyroid scintigram seems to be a simple repetitive task for nuclear medicine physicians, but it is only with a moderate interobserver agreement among endocrinologists [3], there remains an unmet need to assist the reader in analyzing thyroid scintigraphy more consistently and accurately.

Recently, Artificial intelligence (AI) demonstrated distinguished advances in big-data retrieval, explicit feature extraction, satisfactory consistency, and efficiency in terms of medical image analysis [4–6]. It has been proven effective in the analysis of single positron emission computed tomography (SPECT) images. For instance, myocardial perfusion imaging and whole-body bone scan were successfully assessed and reported by implementing deep learning approach [7–9]. A previous study [10] used deep convolutional neural networks (DCNN) with optimization for thyroid diagnosis from SPECT images and reached almost perfect performance in classifying three common thyroid diseases. However, conventional clinical practice considers that diagnosis of thyroid disease is not only based on thyroid scintigrams but with available biochemical data, clinical history, and physical examination [11]. There is still not a one-to-one correspondence between thyroid scintigrams types and specific thyroid disease, since the entirely different thyroid diseases would present similar thyroid scintigrams characteristics [2]. Furthermore, the researches mentioned above omitted one of the most important indications in thyroid scintigraphy, namely the autonomously functioning thyroid nodules which present focal increased uptake in thyroid scintigram [1, 12]. Thus, we input four common thyroid uptake patterns from thyroid scintigrams instead of idiographic thyroid disease to train our AI model and validated the performance on the internal and external datasets in dual centers.

## Methods

### Collection, inclusion, and exclusion of patients

This study with retrospective information collection was approved by the Institutional Ethics Committee of West China Hospital in Sichuan University and Panzhihua Central Hospital, respectively. We retrospectively collected cases who underwent ^99m^Tc-pertechnetate thyroid scintigraphy from January 1, 2016 to December 31, 2018 at West China Hospital of Sichuan University (Center 1) and Panzhihua Central Hospital (Center 2). The patients who were confirmed thyrotoxicosis through clinical history and thyroid function tests (thyroid stimulating hormone, free triiodothyronine, and free thyroxine) were included. The exclusion criteria were listed as following: (1) Patients who underwent semi/total thyroidectomy; (2) failed to extract raw data from picture archiving and communication system (PACS); (3) images format was not raw data; (4) images were incomplete. The thyroid scintigram in two hospitals was obtained following the clinical guidelines and manufacturer recommended parameters. Briefly, patients were intravenously injected with 185 MBq of ^99m^TcO4^−^, and then the images were captured for 100 × 10^3^ counts in 5 min (center 1) and 300 × 10^3^ counts in 10 min (center 2) using the gamma cameras, which were both equipped with the low-energy, high-resolution, parallel-hole collimators (GE Discovery NM/CT 670). And the pixel size, matrix size, and field of view (FOV) were 2.21 mm, 256 × 256, and 28 cm in center 1, which is 2.21 mm, 128 × 128, and 28 cm in center 2, respectively. The energy peak was centered at 140 keV with 15–20% windows. All the images were exported as DICOM format for further analysis.

### Classification criteria

**Fig. 1:**
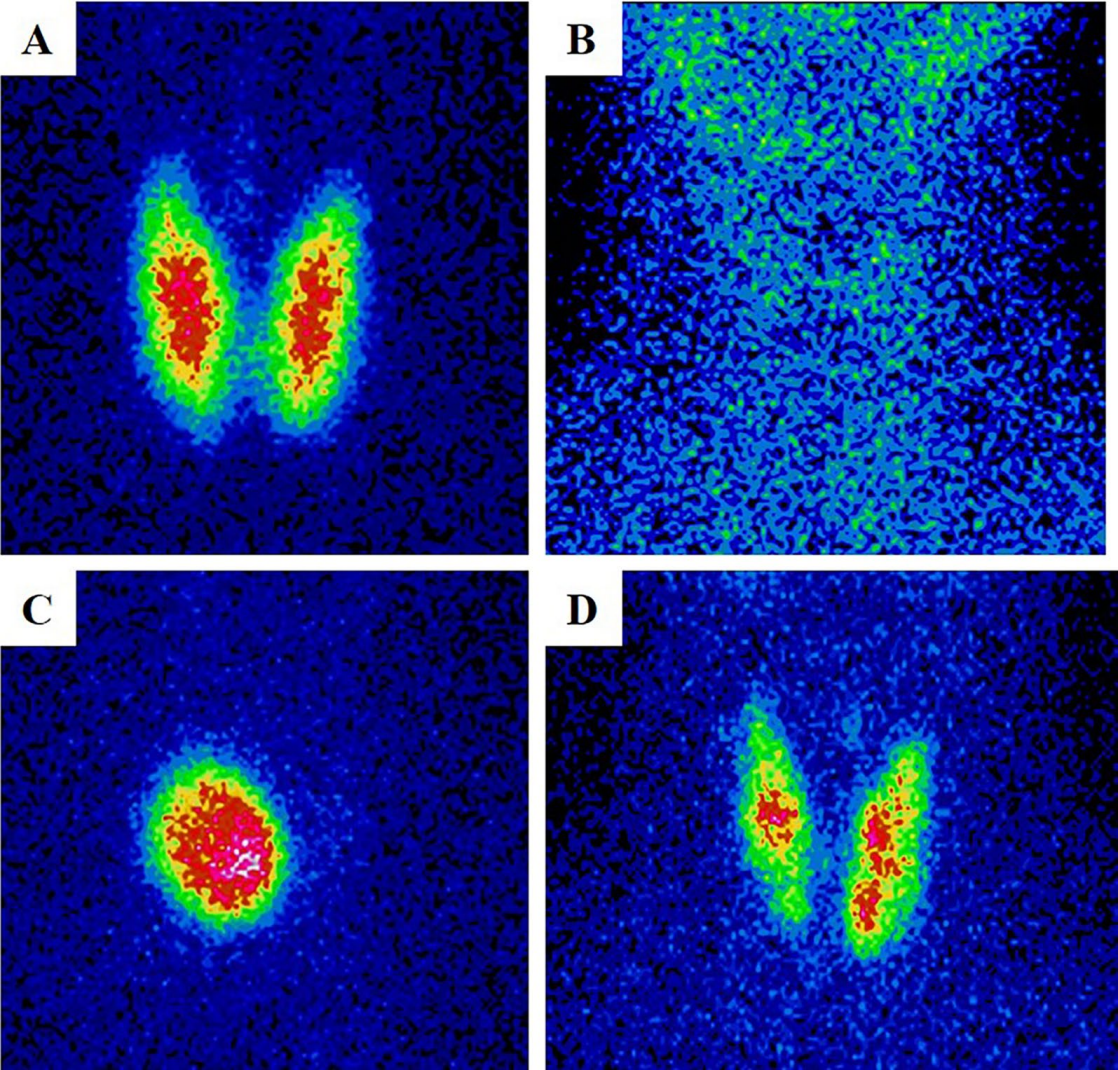
The characteristic performance of ‘diffusely increased’ (**A**), ‘diffusely decreased’ (**B**), ‘local increased’ (**C**) and ‘heterogeneous uptake’ (**D**)

Thyroid scintigrams were defined as four common patterns referring to published criteria [2, 11, 13, 14]. The ones that had homogeneous increased uptake over than the uptake of salivary with enlarged thyroid were defined as ‘Diffusely increased’ (type I); the ones that had diminished and absent uptake was defined as ‘diffusely decreased’ (type II); the ones had focal nodule uptake with or without suppressed uptake in the surrounding thyroid tissue was defined as ‘local increased’ (type III), and the ones had multiple areas of focal increased and suppressed uptake was defined as ‘heterogeneous uptake’ (type IV). All characteristic performance of these four pattern images were shown in Fig. 1. For this study, all thyroid scintigram images from two centers were independently and blindly classified by three senior nuclear medicine physicians with more than 10 years of working experience in reading thyroid scintigraphic images. Consensus shall be reached by consulting if there is disagreement.

### Construction of AI model

**Fig. 2:**
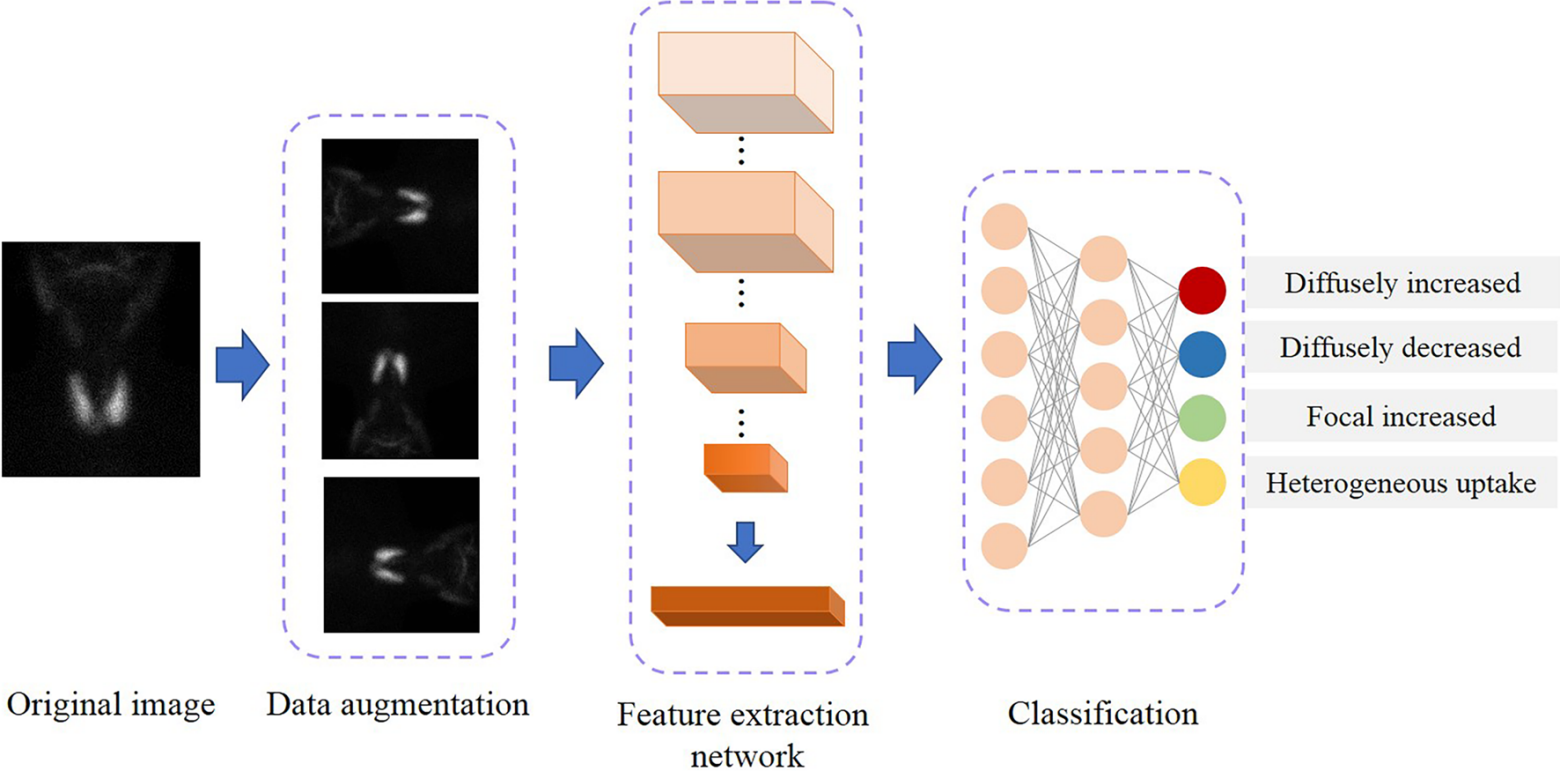
The architecture process of AI model

The images collected from center 1 were defined as the internal dataset for AI construction and internal validation, while the images from center 2 were defined as the external dataset for validation only. The architecture of the AI model is illustrated in Fig. 2. There are three main steps in the training process: data augmentation, feature extraction, and classification. Before data augmentation, all images were converted to grayscale images with a value range of [0,255] according to the range of intensity. Random horizontal flipping with a probability of 0.5, random rotation by 0°–90°, and mix-up [15] were applied to the original image to increase the diversity of the data and improve the robustness of the model in augmentation. After data augmentation, those images were normalized by divided 255. Then, a feature extraction neural network is employed to extract high-level features from the input image. The feature extraction neural network is consist of various layers including convolutional, batch normalization, pooling, and ReLU layers. In this study, we explored four kinds of candidate AI models based on different standout pre-trained networks, including ResNet50 [16], DenseNet169 [17], InceptionV3 [18], and InceptionResNetV2 [19]. All these networks have been removed the last fully connected layer and employed as the feature extraction network. At the final step, a neural network that contains three fully connected layers is constructed to classify the high-level features into four classes. In the current study, all models were trained using Adam [20] as the optimizer with a weight decay rate of 0.0001 and a learning rate of 0.001 for 300 epochs. The mini-batch size was fixed 12. To reduce overfitting's side effect, we employed the dropout [21] to the last fully connected layer, with a drop probability of 0.8.

### Evaluation of model performance

The classification accuracy, sensitivity, specificity, positive predictive value (PPV), negative predictive value (NPV), precision, recall, and F1 score of four candidate DCNNs were individually evaluated in the internal and external validation. True positive (TP), true negative (TN), false positive (FP) and false negative (FN) can be determined for each category according to whether the classification results of DCNNs are correct and whether the samples are positive. The mathematical formulations of the above evaluation metrics were defined as follows:

$$Accurracy=\frac{\mathrm{TP}+\mathrm{TN}}{TP+FP+TN+FN}$$

$$Sensitivity (Recall)=\frac{\mathrm{TP}}{TP+FN}$$

$$Specificity=\frac{\mathrm{TN}}{TN+FP}$$

$$PPV (Precision)=\frac{TP}{TP+FP}$$

$$NPV=\frac{\mathrm{TN}}{TN+FN}$$

$$F1 score=2\times \frac{\mathrm{Recall}\times \mathrm{Precision}}{Recall+Precision}$$

The larger these performance values are, the better the performance of a method is. Then, the performance of four DCNNs in the internal and external validation was evaluated by areas under the curve (AUC) of receiver operating characteristic (ROC) as well. The 4 × 4 confusion matrix visualized the classification true labels and predicted labels of four DCNNs in identifying four thyroid uptake patterns from thyroid scintigrams.

## Results

### Patient characteristics

We collected 2468 cases of thyroid scintigrams (2396 females and 72 males; age: 41.24 ± 14.25 years) as a training cohort and 619 cases (611 females and 8 males; age: 41.20 ± 14.20 years) as an internal validating cohort from West China Hospital of Sichuan University (center 1). Another 302 cases (214 females and 88 males; age: 44.61 ± 13.68 years) were obtained from Panzhihua Central Hospital (center 2) as an external validating cohort. In center 1, ‘diffusely increased’ and ‘diffusely decreased’ predominated, whereas ‘diffusely increased’ and ‘heterogeneous uptake’ accounted for the majority in center 2. Furthermore, the ‘focal increased’ was relatively deficient in both centers. The detailed distribution of thyroid scintigrams at dual centers was shown in Table 1.

**Table 1:** The detailed distribution of thyroid scintigrams at dual centers

Source	Dataset	Diffusely increased	Diffusely decreased	Focal increased	Heterogeneous uptake	Total
Training	Internal	1040 (42.14)	902 (36.55)	97 (3.93)	429 (17.38)	2468
Validating	Internal	260 (42.00)	226 (36.51)	25 (4.04)	108 (17.45)	619
	External	120 (39.74)	49 (16.22)	13 (4.30)	120 (39.74)	302

Values are described as absolute numbers or percentages in the parantheses

### Performance of the DCNNs

**Fig. 3:**
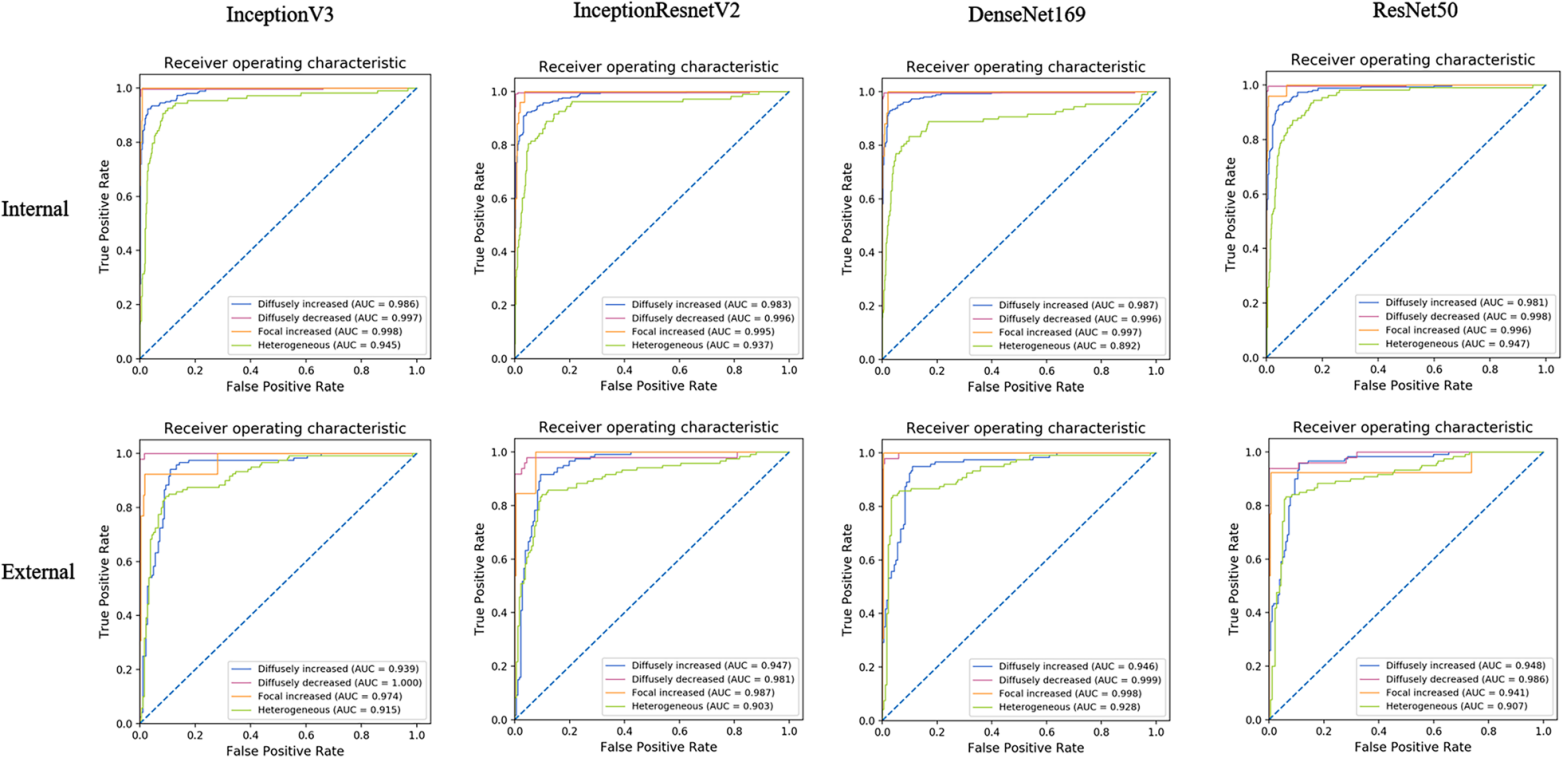
The performance of four DCNNs by using AUC calculation in classifying four patterns of thyroid scintigrams in the internal and external validation

**Fig. 4:**
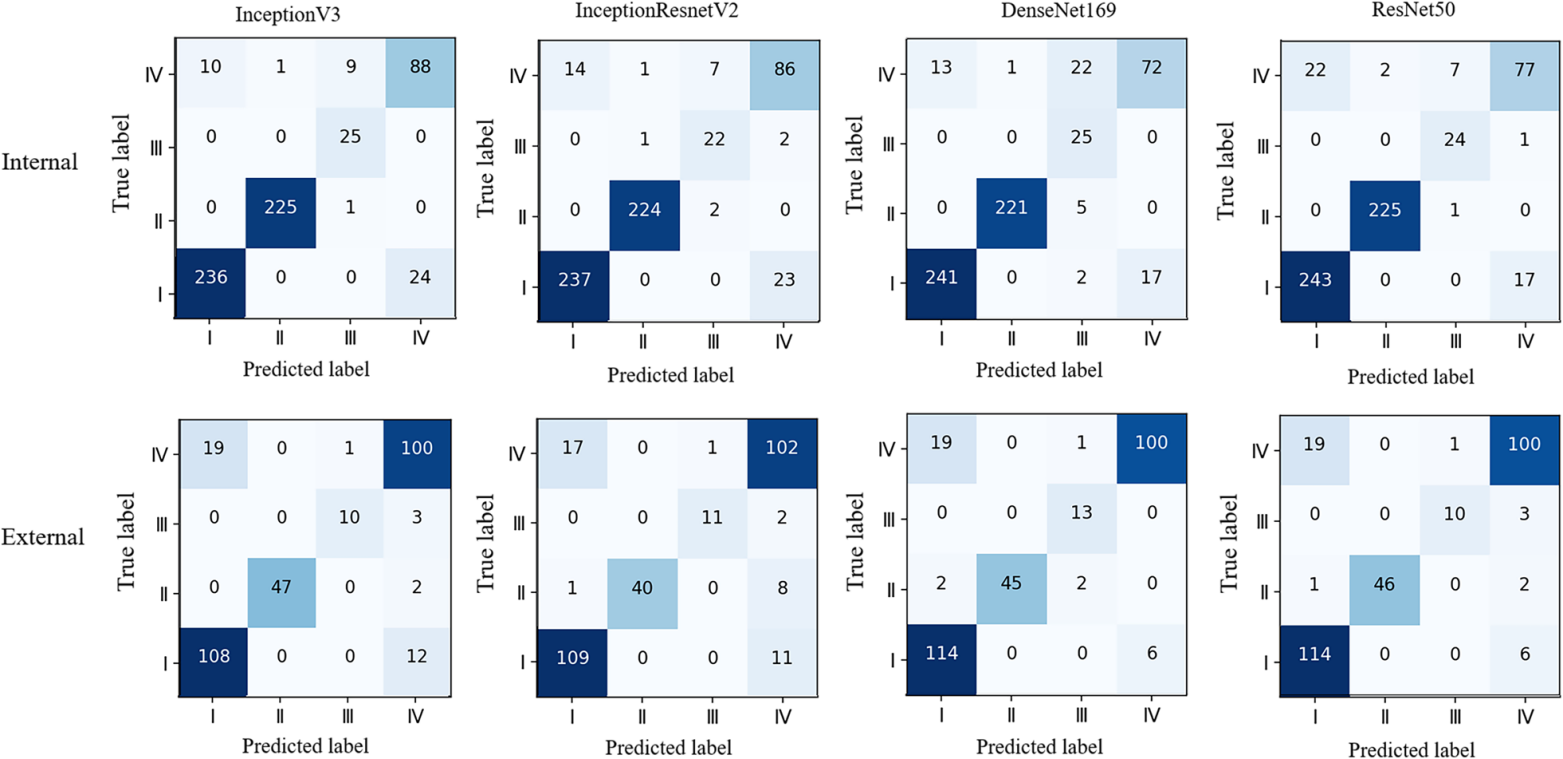
The confusion matrix of four DCNNs in classifying four patterns of thyroid scintigrams in the internal and external validation. Type I: diffusely increased; Type II: diffusely decreased; Type III: local increased; Type IV: heterogeneous uptake

The individual performances of four DCNNs in internal and external validation were shown in Table 2. The InceptionV3 model achieved the highest overall accuracy of 92.73% (574/619) in classifying four common patterns of thyroid scintigrams in the internal validation, whereas the metrics dropped to 87.75% (265/302) in external validation. After applied ROC, the AUC values of the InceptionV3 in the diagnosing of four thyroid uptake patterns reached a considerable performance, which the AUC was 0.986 for ‘diffusely increased,’ 0.997 for ‘diffusely decreased,’ 0.998 for ‘focal increased,’ and 0.945 for ‘heterogeneous uptake’ in internal validation, respectively. Accordingly, the corresponding performances also obtained an ideal result of 0.939, 1.000, 0.974, and 0.915 in external validation, respectively. The confusion matrix demonstrated that the recall of the InceptionV3 reached a profitable result, which is 90.77% (236/260) for ‘diffusely increased,’ 99.56% (225/226) for ‘diffusely decreased,’ 100.00% (25/25) for ‘focal increased’ in the internal validation. Whereas, the recall for ‘heterogeneous uptake’ was relatively moderate, which is 81.48% (88/108). The category of ‘heterogeneous uptake’ was more likely to be misclassified into ‘diffusely increased’. In the external validation, the selected DCNN displayed comparable performance in the recognizing of ‘diffusely increased’, ‘diffusely decreased’ and ‘heterogeneous uptake’. But for the category of ‘focal increased, the recall dropped significantly to 76.92% (10/13). The results of the ROC analysis (Fig. 3) and the confusion matrix (Fig. 4) of the other three DCNNs are listed as well.

**Table 2:** The performance of DCNNs that including InceptionV3, InceptionResnetV2, DenseNet169, and ResNet50 in the internal and external datasets

Metrics	Dataset	Diffusely increased (%)	Diffusely decreased (%)	Focal increased (%)	Heterogeneous uptake (%)
*InceptionV3*					
Accuracy	Internal	94.51	99.68	98.38	92.89
	External	89.74	99.34	98.68	87.75
Sensitivity (Recall)	Internal	90.77	99.56	100.00	81.48
	External	90.00	95.92	76.92	83.33
Specificity	Internal	97.21	99.75	98.32	95.30
	External	89.56	100.00	99.65	90.66
PPV(Precision)	Internal	95.93	99.56	71.43	78.57
	External	85.04	100.00	90.91	85.47
NPV	Internal	93.57	99.75	100.00	96.06
	External	93.14	99.22	98.97	89.19
F1 score	Internal	93.28	99.56	83.33	80.00
	External	87.45	97.92	83.33	84.39
*InceptionResnetV2*					
Accuracy	Internal	94.02	99.35	98.06	92.41
	External	90.40	97.02	99.01	87.09
Sensitivity (Recall)	Internal	91.15	99.12	88.00	79.63
	External	90.83	81.63	84.62	85.00
Specificity	Internal	96.10	99.49	98.48	95.11
	External	90.11	100.00	99.65	88.46
PPV(Precision)	Internal	94.42	99.12	70.97	77.48
	External	85.83	100.00	91.67	82.93
NPV	Internal	93.75	99.49	99.49	95.67
	External	93.71	96.56	99.31	89.94
F1 score	Internal	92.76	99.12	78.57	78.54
	External	88.26	89.89	88.00	83.95
*DenseNet169*					
Accuracy	Internal	94.83	99.03	95.32	91.44
	External	91.06	98.68	99.01	91.39
Sensitivity (Recall)	Internal	92.69	97.79	100.00	66.67
	External	95.00	91.84	100.00	83.33
Specificity	Internal	96.38	99.75	95.12	96.67
	External	88.46	100.00	98.96	96.70
PPV(Precision)	Internal	94.88	99.55	46.30	80.90
	External	84.44	100.00	81.25	94.34
NPV	Internal	94.79	98.74	100.00	93.21
	External	96.41	98.44	100.00	89.80
F1 score	Internal	93.77	98.66	63.29	73.10
	External	89.41	95.74	89.66	88.50
*ResNet50*					
Accuracy	Internal	93.70	99.52	98.55	92.08
	External	91.39	99.01	98.68	89.74
Sensitivity (Recall)	Internal	93.46	99.56	96.00	71.30
	External	95.00	93.88	76.92	83.33
Specificity	Internal	93.87	99.49	98.65	96.48
	External	89.01	100.00	99.65	93.96
PPV(Precision)	Internal	91.70	99.12	75.00	81.05
	External	85.07	100.00	90.91	90.09
NPV	Internal	95.20	99.74	99.83	94.08
	External	96.43	98.83	98.97	89.53
F1 score	Internal	92.57	99.34	84.21	75.86
	External	89.76	96.84	83.33	86.58

## Discussion

Thyroid scintigraphy is a convenient and intuitive technology in evaluating the distribution of active thyroid tissue in clinical practice. It provides a clue to distinguish the causes of thyrotoxicosis by corresponding with four common uptake patterns [11, 22, 23]. However, inevitable variations still exist among different inter-observers in interpreting thyroid scintigram [3]. If physicians interpret the same thyroid scintigram differently, different treatments might be recommended in further clinical management. Considering the distinguished advances of DCNN in explicit feature extraction and satisfactory consistency in medical data analysis, we tried to construct an AI model to help physicians interpret thyroid scintigrams.

Overall, this AI model indicated a satisfactory classification performance. The accuracy of distinguishing four common thyroid uptake patterns from thyroid scintigrams in the internal validation was 92.73% and 87.75% in the external validation, respectively. Slightly declined accuracy was observed when applied the DCNN to the new dataset of “high-signal abundant images” with 300 × 10^3^ counts. Although there is an acquisition guideline for thyroid scintigraphy [1], imperceptible divergences have still existed in images obtained by different institutes, equipment, or under different system parameters, and afterward, these variations may accumulate and affect the final interpretation of thyroid scintigrams. The relationship between acquisition variations in the training cohort is worth considering. Furthermore, the model had high advantages in the recognition of ‘diffusely increased,’ and ‘diffusely decreased,’ in dual centers. But the performance for the ‘heterogeneous uptake’ pattern was relatively low in internal and external validation, and we found that this thyroid uptake pattern was preferred to be misclassified into ‘diffusely increased’. We presumed it is due to the suppressed uptake feature could not be extracted well as increased uptake by the DCNN.

The sensitivity of our model is slightly lower compared with Ma et al. [10], which the sensitivity almost reached 100% in classifying GD (97.5%), Hashimoto disease (98.5%), subacute disease (100%), and normal class (100%). This discrepancy could due to the diverse datasets, it was better to include normal thyroid images to deep learning for distinguishing abnormal thyroid disease. However, we input and output four common thyroid uptake patterns according to the physician’s interpretation, rather than input specific thyroid disease. As widely regarded in clinical practice, some thyroid diseases could share a similar uptake pattern in thyroid scintigraphy, such as endemic goiter Hashimoto’s thyroiditis and Graves’ disease [2, 24, 25]. Thus, directly output the specific thyroid disease prefers to increase the risk of misdiagnosis. On the contrary, automatically recognize and distinguish thyroid uptake patterns in thyroid scintigraphy potentially facilitates the consistency and efficiency of interpretation of thyroid scintigrams, especially for practicing physicians.

Nevertheless, we also noticed some unsatisfied points in this study. Firstly, the model’s performance was found not as good as in the external validation, which encourages the necessity to enroll a larger dataset from multi-institutes to facilitate a new model with better serviceability in available generalization. Then, as we discussed above, thyroid scintigram is not sufficient to accomplish the diagnosis of thyroid disease, a new robust model that could analyze multi-type data is under development. We believe that AI-assisted diagnosis would be more precise for specific thyroid diseases by integrating clinical history, biochemical information, and thyroid scintigrams.

## Conclusion

We have successfully constructed an AI model for classifying four common patterns of thyroid scintigrams and achieved considerable accuracy in dual centers. With further assessment and validation, this model might be promising in the clinical interpretation of thyroid scintigraphy in thyrotoxicosis.

## Data Availability

The datasets generated and analyzed during the current study are not publicly available but available from the corresponding author upon reasonable request.

## Abbreviations

AI: Artificial intelligence
DCNN: Deep convolutional neural networks
GD: Graves’ disease
TMG: Toxic multinodular goiter
PPV: Positive predictive value
NPV: Negative predictive value
AUC: Areas under the curve
ROC: Receiver operating characteristic
